# Case Report: Multimodality Imaging of Infectious Sacroiliitis and Retroperitoneal Abscess Causing Hindlimb Ataxia in a Young Dog

**DOI:** 10.3389/fvets.2021.732788

**Published:** 2021-10-15

**Authors:** Jeongyun Jeong, Jaeeun Ko, Jaehwan Kim, Kidong Eom, Youngkwon Cho, Kichang Lee, Hakyoung Yoon

**Affiliations:** ^1^Department of Veterinary Medical Imaging, College of Veterinary Medicine, Konkuk University, Seoul, South Korea; ^2^College of Health Sciences, Cheongju University, Cheongju, South Korea; ^3^Department of Veterinary Medical Imaging, College of Veterinary Medicine, Jeonbuk National University, Iksan-si, South Korea

**Keywords:** infectious sacroiliitis, osteomyelitis, computed tomography, ultrasonography, magnetic resonance imaging

## Abstract

A 3-month-old intact male Labrador Retriever was presented for falling trauma and hindlimb ataxia. Radiography indicated radiolucent left sacroiliac joint with irregular margin. Computed tomography revealed thickened sublumbar muscles and hypoattenuated sacroiliac joint while magnetic resonance imaging demonstrated abscess at retroperitoneum and gluteal muscle. Ultrasonography showed lytic left sacroiliac joint with retroperitoneal fluid, and fine needle aspiration resulted *Staphylococcus aureus*. Hindlimb ataxia was attributed to infectious sacroiliitis and its secondary retroperitoneal abscess. As far as the authors' knowledge, this is the first report of multimodality imaging of infectious sacroiliitis with retroperitoneal abscess caused by *S. aureus* in a dog.

## Introduction

Infectious sacroiliitis is a rare disease in human and veterinary medicine and accounts for 1–2% of septic arthritis in humans (1, 2). *Pasteurella* and *Brucella* have been reported as causative agents in veterinary literatures, but *Staphylococcus aureus* is the most common agent in humans (1, 3). Abscess formation has been reported as a secondary result of infectious sacroiliitis, but those caused by *S. aureus* have not been reported in veterinary medicine (1). Thus, this report aims to introduce multimodality imaging findings of infectious sacroiliitis and retroperitoneal abscess caused by *S. aureus* in a young dog.

## Case Description

A 3-month-old intact male Labrador Retriever was referred because of recent falling trauma and subsequent hindlimb weakness, depression, and anorexia. The referring veterinarian suspected microfracture of the sacrum and soft tissue contusion. On physical examination, palpation and maneuvering of hindlimbs elicited pain response. Gait analysis revealed paresis of both bilateral hindlimbs to supplement clinical findings. Neurologic examination indicated intact deep pain response of the hindlimbs but diminished proprioceptive positioning and absence of hopping. Spinal reflexes of both hindlimbs, including quadriceps, extensor carpi, and flexion, were all reduced as well. Cranial nerves examination and mental status were unremarkable. Complete blood count analysis revealed leukocytosis (27,000/μl; reference range 5,050–16,760/μl) and high C-reactive protein level (138 mg/L; reference range 0–20). Along with toxic changes of leukocytes, many juvenile leukocytes and band cells were noted in the blood smear examination.

**Figure 1:**
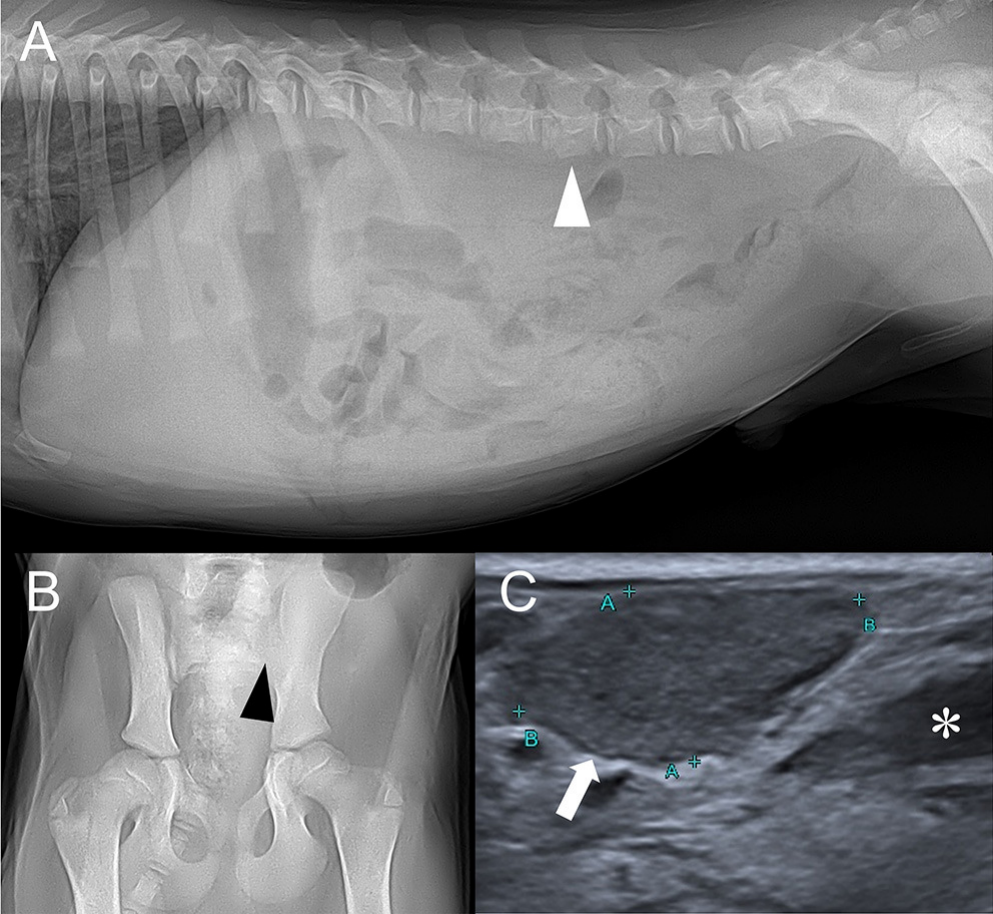
Right lateral abdominal **(A)**, ventrodorsal coxofemoral joint **(B)** radiographs, and abdominal ultrasound depicting sublumbar lymph node **(C)**. **(A)** Irregular ventral margin of the 4th lumbar vertebra (white arrowhead) and decreased serosal detail are found. **(B)** Left sacroiliac joint is radiolucent and its bone margin (black arrowhead) is indistinct. **(C)** Enlarged sublumbar lymph node (white arrow) and anechoic trapped retroperitoneal fluid (asterisk) are noted.

Thoracic radiography (Titan 2000M, COMED Medical System, Seoul, Korea) was unremarkable. However, abdominal radiography revealed irregular ventral margin of 4th lumbar vertebra and decreased abdomen serosal detail (Figure 1A). Young age was the likely cause of the decreased serosal detail, but possibility of peritonitis, retroperitonitis, and ascites existed (4). Irregular margin of the lumbar vertebra raised concerns for previous vertebral physeal fracture, which could have damaged adjacent spinal cord. Additionally, coxofemoral joint radiography revealed radiolucent left sacroiliac joint with indistinct bone margins, indicating bone lysis (Figure 1B). Considering the short time between the initial traumatic event and the hospital referral, diffuse bone lysis of sacroiliac joint was unlikely to be caused by the trauma. Instead, based on the patient's radiographic findings, differential diagnosis for sacroiliac joint included infectious sacroiliitis, non-infectious sacroiliitis and less likely joint tumor.

Abdominal ultrasound performed (Aplio 300, Toshiba Medical System, Tokyo, Japan) with linear-array (10 MHz) probe revealed bilateral sublumbar and inguinal lymphadenopathy (Figure 1C). Moderate amount of trapped anechoic fluid was found in the retroperitoneal space, adjacent to enlarged sublumbar lymph nodes. Also, retroperitoneum was thickened and edematous along with diffusely hyperechoic adjacent muscles. There were no remarkable findings of urinary tract. Based on the ultrasonographic findings, retroperitonitis with adjacent myositis was suspected.

**Figure 2:**
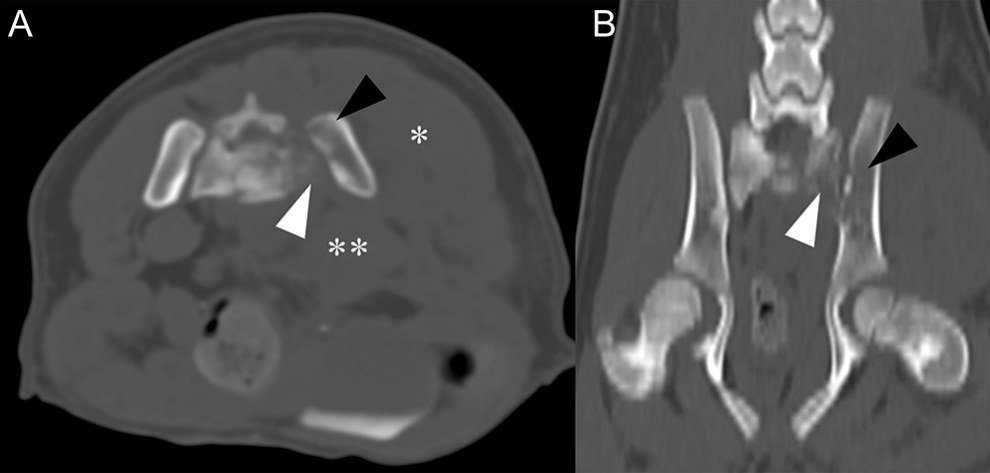
Transverse plane, pre-contrast CT image **(A)** and dorsal plane, pre-contrast CT image **(B)** in bone algorithm at the level of sacroiliac joint level. **(A)** Compared to right sacrum and ilium, left sacrum (white arrowhead), and ilium (black arrowhead) have relatively low attenuation and indistinct bone margins. Left gluteal (asterisk) and sublumbar muscle (double-asterisk) show marked swelling and increase in thickness. Note widening of retroperitoneal space. **(B)** Low attenuation of left sacrum (white arrowhead) and ilium (black arrowhead) are noted.

To further characterize the bone lysis of sacroiliac joints and the irregular margin of the lumbar 4th vertebra, computed tomography (CT) examination was performed (SOMATOM, Siemens Healthcare, Erlangen, Germany). Imaging parameters were slice thickness 2 mm, pitch 0.8, tube rotation time 0.5 s, 130 kVp, 100 mAs, 512 × 512 matrix. Left sacral bone and ilium exhibited lower bone attenuation compared to the right sacral and ilium (Figure 2). Left sublumbar muscles, including psoas major and psoas minor, were edematous and thickened (Figure 2A). Also, hypoattenuating fluid (HU = 18) with indistinct margin was found medial to the left psoas muscle at the level of sacroiliac joint. These findings contributed to the widening of retroperitoneal space. Left gluteal muscle was also thickened. Based on the CT findings, differential diagnosis for left sacroiliac joint included osteomyelitis of the left sacrum and ilium along with myositis of left sublumbar and gluteal muscle. Together with ultrasonographic findings, retroperitoneal abscess and hemorrhage were suspected. Protuberance of ventral margin of the lumbar 4th vertebra was noted, but no clear signs of fracture was seen. Therefore, normal variant shape of the lumbar vertebrae was considered.

**Figure 3:**
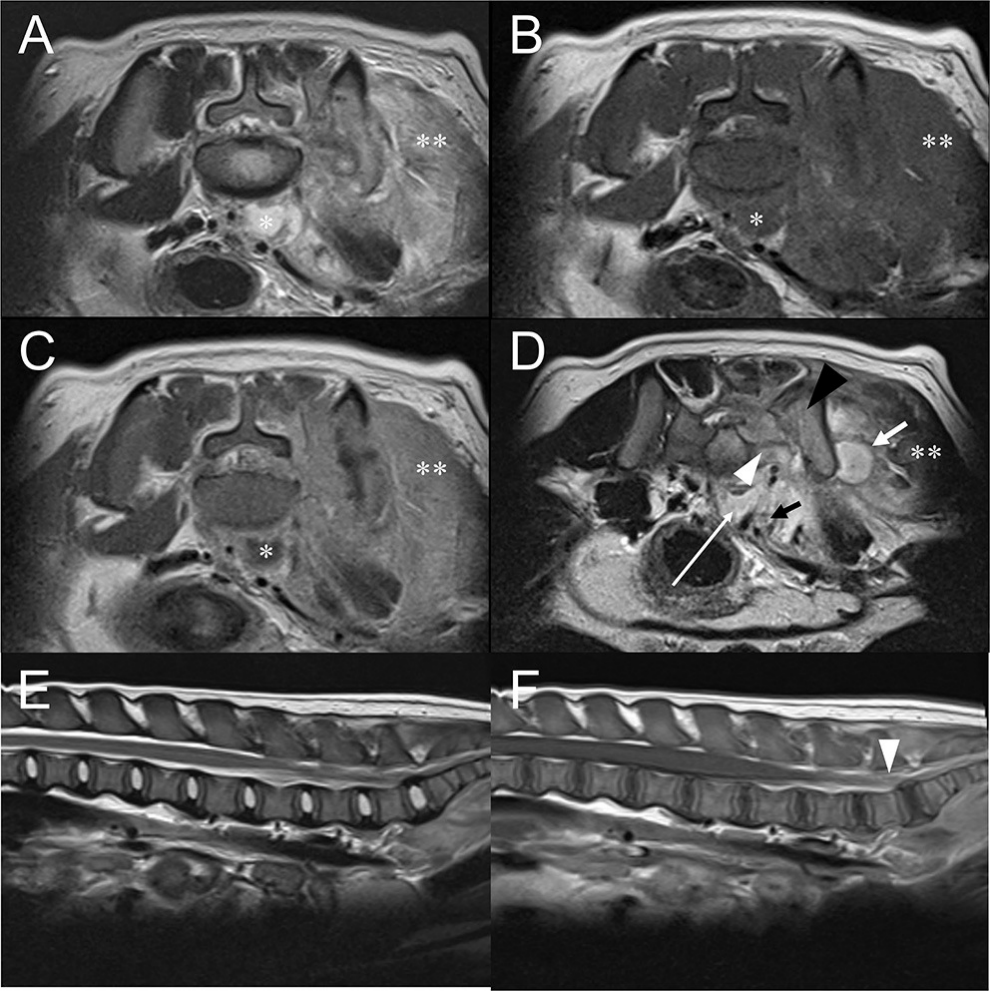
Transverse T2W image **(A)**, pre-contrast T1W image **(B)**, post-contrast T1W image **(C)** at the level of L7-S1 intervertebral space and T2W image **(D)** at the level of sacroiliac joint. Sagittal T2W image **(E)** of the lumbar spinal cord and post-contrast T1W images **(F)** of the lumbar spinal cord. **(A–C)** T2W hyperintense, T1W hypointense, and contrast-enhanced T1W rim enhancing structure (asterisk) located at ventral of the lumbar vertebral body represents retroperitoneal abscess. **(D)** Retroperitoneal abscess (white long arrow) is found at the level of sacroiliac joint. Muscular abscess (white short arrow) is also indicated. Compared to the right sacral bone, left sacral (white arrowhead) and iliac bone (black arrowhead) are T2W hyperintense and indistinct left sacroiliac joint space, which is consistent with sacroiliitis. Left gluteal, sacrocaudal, and sublumbar muscle (double asterisk) show marked swelling, T2W hyperintense, and enhanced on contrast-enhanced T1W. **(E)** No abnormality is noted in the lumbar spinal cord of the T2W sagittal image. **(F)** Compared to other regions of lumbar spinal cord, mildly increased intensity of the spinal cord at the level of L7-S1 is shown (arrowheads) at post-contrast T1W. This could represent true enhancement but also could be due to slice thickness artifact.

Magnetic resonance imaging (MRI) examination of the lumbar spinal cord was performed with 1.5 T scanner (Magnetom Essenza, Siemens Healthcare, Erlangen, Germany). The study included T2-weighted (T2W) images in sagittal and transverse planes (TR 2,200–5,830 ms, TE 113–116 ms), pre- and postcontrast T1-weighted (T1W) images in sagittal and transverse planes (TR 470–589 ms, TE 13 ms), and gradient echo (T2^*^) images in transverse plane (TR 410 ms, TE 20 ms, flip angle 20 degrees). Slice thickness was 3–4 mm and intervals were 0.3–0.85 mm. Post-contrast T1W images were acquired after administrating 0.15 mmol/kg of gadolinium-based contrast agent intravenously (Magnevist, Bayer, NJ, USA). No remarkable changes were noted in lumbar spinal cord on T2W images (Figure 3D) but intensity of the spinal cord at the level of L7-S1 was mildly increased on post-contrast T1W images (Figure 3F). Hyperintense left sacrum and ilium on T2W images and indistinct left sacroiliac joint space were noted (Figure 3D). Also, ill-defined and thickened gluteal muscle appeared hyperintense on T2W images and strongly enhanced on T1W contrast-enhanced images (Figures 3A,C). Retroperitoneal space at level of lumbosacral joint exhibited T1W hypointense, T2W hyperintense, contrasted-enhanced T1W rim enhancing hypointense lesion with distinct margin (Figure 3). Similar lesions were also found at muscles lateral to left ilium. These findings indicated retroperitoneal and muscular abscess. Presence of retroperitoneal abscess raised a possibility of adjacent lumbosacral plexus compression, inflammation, or infection. Spinal tap was not performed because owner refused it. Based on the MRI findings, tentative diagnosis included sacroiliac joint osteomyelitis, left gluteal muscle myositis and abscess, and retroperitoneal abscess with likelihood of peripheral neuropathy of lumbosacral plexus.

**Figure 4:**
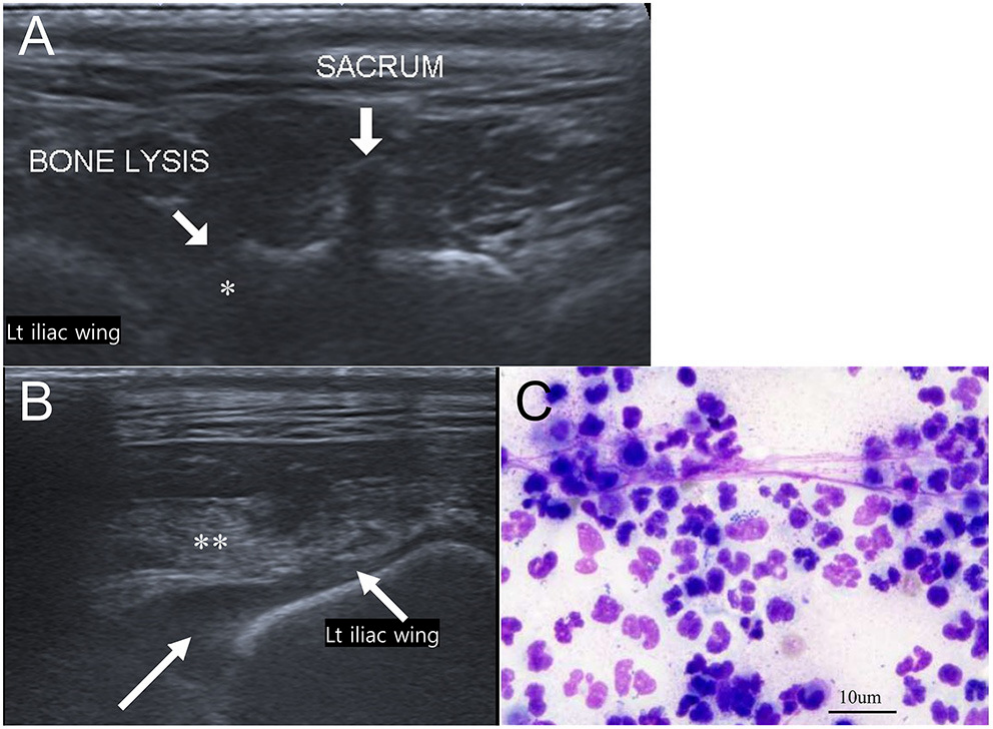
Musculoskeletal ultrasound images of the sacroiliac joint **(A,B)** and cytologic evaluation of aspirated trapped fluid around the iliac wing (Wright-Giemsa stain, ×400 magnification) **(C)**. **(A)** Adjacent to left iliac wing, lytic changes of left sacral bone (asterisk) is noted. **(B)** Trapped anechoic fluid (white long arrow) and hyperechoic muscle (double asterisk) are found in the retroperitoneum near left iliac wing. **(C)** Cytologic evaluation indicates numerous degenerated neutrophils and phagocyted cocci.

Musculoskeletal ultrasound with linear-array (10 MHz) probe was performed to evaluate the bone margins of the sacroiliac joints and soft tissue changes. Bone lysis of the left sacrum and the iliac wing was noted (Figure 4A). Anechoic trapped fluid and hyperechoic muscles adjacent to iliac wing were also observed, which indicated myositis (Figure 4B). Ultrasound-guided fine needle aspiration (FNA) of the fluid adjacent to iliac wing and retroperitoneal fluid was performed. Pyogenic fluid was aspirated and cytologic examination demonstrated myeloblast, numerous degenerated neutrophils, and phagocyted cocci (Figure 4C). FNA was also performed in the inguinal and sublumbar lymph nodes, with the latter exhibiting many neutrophils and some phagocyted cocci. The aspirated materials were sent for antimicrobial analysis and antibiotic susceptibility test.

Considering the findings of the imaging studies and the cytologic evaluation, infectious sacroiliitis, myositis, and retroperitoneal abscess were likely. To prevent sepsis, antibiotic therapy was warranted. While pending for the antibiotic susceptibility test result, the patient started with cefazolin (30 mg/kg, IV, QID), famotidine (0.5 mg/kg IV, BID), and fluid therapy (Hartmann solution, 5 ml/kg/h), which dramatically reduced leukocytosis and C-reactive protein level. Antimicrobial analysis indicated *Staphylococcus aureus* and cefazolin to be effective against the microorganism. After antimicrobial treatment, the patient showed improved appetite and hindlimb condition. The patient still showed some hindlimb ataxia but was able to support his body weight with his legs and ambulate. Due to the owner's request, the patient was discharged. At 1 week follow-up after the diagnosis, the patient started rehabilitation therapy along with antimicrobial treatment. Prognosis of the patient was regarded as very good because the patient's condition improved dramatically 3 days after the antibiotic treatment and was able to ambulate 1 week after. At 1 month follow-up after the diagnosis, the patient did not exhibit hindlimb ataxia.

## Discussion

This report presents hindlimb ataxia and neurologic deficits in patients with retroperitoneal abscess and infectious sacroiliitis. The abscess in the retroperitoneal space was secondary to sacroiliitis along with retroperitonitis. Also, infectious sacroiliitis caused gluteal and sublumbar muscle myositis. These findings could have all contributed to hindlimb ataxia.

In veterinary medicine, few literatures regarding infectious sacroiliitis exist. A study reported diagnostic imaging of two patients diagnosed with infectious sacroiliitis (1). The authors of the following study reported irregular joint space, contrast enhancement of articular joint structures, increased joint fluid, bone marrow edema, and peri-articular soft tissue edema as findings of infectious sacroiliitis (1). *Pasteurella canis* and *Brucella canis* have been reported as causative agents (1, 3). However, this case presents infectious sacroiliitis by *S. aureus*. In humans, non-iliac dominant pattern of bone marrow edema, large bone erosion, thick capsulitis, extracapsular fluid, and peri-articular muscle edema have been attributed to characteristics of infectious sacroiliitis (2, 5, 6). Hematogenous spread is a well-known route of infection and *S. aureus* is the most common causative agent (7, 8).

Numerous causes can be attributed to hindlimb ataxia but when considering the physical and neurological examination results, abnormalities in lumbar spinal cord or peripheral nervous system could be responsible for the ataxia. Mildly increased intensity of the spinal cord at the level of L7-S1 post-contrast T1W images could represent true enhancement but slice thickness artifact could be responsible for such changes as well. Also, the patient did not exhibit clinical signs attributed to lesions in L7-S1, such as urinary incontinence or dyschezia so lumbar spinal cord lesions at L7-S1 were unlikely. Instead, the authors focused on peripheral nervous system, including lumbosacral plexus because it could be affected as well since inflammation in adjacent retroperitoneum was evident. In this patient, spread of inflammation to retroperitoneum from sacroiliitis resulted in retroperitoneal abscess and edematous, thickened sublumbar muscles. There have been few reports in veterinary literature regarding retroperitoneal abscess with hindlimb gait abnormalities but its relationship between lumbosacral plexus was not discussed (9, 10). Lumbar plexus innervates cranial and medial part of thigh muscles while sacral plexus innervates caudal part of thigh muscles (11). In humans, the term “lumbosacral plexopathy” indicates diseases or disorders affecting the pathway on lumbosacral plexus and numerous etiologies are included (12). Vascular, trauma, compressive, idiopathic, hereditary, infectious, and iatrogenic causes are possible and retroperitoneal abscess could be one of the compressive or inflammatory causes (13). Although retroperitoneal abscess associated with the lumbosacral plexus has not been well-reported in veterinary medicine, peripheral neuropathy by sciatic nerve invasion or compression by retroperitoneal sarcomas (14, 15) has been reported and retroperitoneal abscess could affect the lumbosacral plexus in a similar manner. Histopathological examination of the plexus, which is a limitation of the study, would have revealed the relationship between the abscess and the lumbosacral plexus. Also, electrophysiological studies, including nerve conduction and action potential studies, could have demonstrated peripheral neuropathy in nerves associated with the lumbosacral plexus. Thus, a lack of electrophysiological studies is a limitation as well.

Absence of spinal tap is another limitation. Spinal tap result would have provided additional information about the spinal cord. Unfortunately, the owner refused it, so it was not performed. Lack of short tau inversion recovery (STIR) or fat-suppressed sequence of MRI demonstrating abscess is another limitation of this study. Nonetheless, recognition of fluid in the retroperitoneal space and successful FNA under ultrasonography guidance led authors to correctly identify retroperitoneal abscess.

In some private practices, not all imaging modalities mentioned in this study are available. All four modalities (radiography, ultrasonography, CT, and MRI) contribute to the diagnosis but some could be unnecessary. Radiography and ultrasonography are necessary to visualize the bone lysis and adjacent retroperitoneal changes. MRI is also indispensable to rule in/out spinal cord diseases. However, CT could be unnecessary since bone lysis could be depicted in radiography and retroperitoneal abscess in ultrasonography or MRI.

## Concluding Remarks

In conclusion, infectious sacroiliitis and retroperitoneal abscess should be considered when a young dog with hindlimb ataxia and neurologic abnormalities exhibits lytic sacroiliac joint with retroperitoneal fluid with adjacent inflammation. To the authors' knowledge, this is the first report to demonstrate multimodality imaging of infectious sacroiliitis and retroperitoneal abscess caused by *S. aureus*. Ultrasound-guided FNA of sacroiliac joint is a powerful technique that allows correct diagnosis of infectious origin and is strongly recommended.

## Data Availability Statement

The original contributions presented in the study are included in the article/supplementary material, further inquiries can be directed to the corresponding author.

## Ethics Statement

Ethical review and approval were not required for the animal study because this was a retrospective case report. Written informed consent was obtained from the owner for the publication.

## Author Contributions

JJ and HY contributed to manuscript writing and image interpretation. HY contributed to radiography, ultrasonography, CT, and MRI scanning. JKo, JKi, and KE assisted image interpretation. YC, KL, JJ, and HY contributed to manuscript editing. All authors reviewed and approved the final submitted manuscript.

## Conflict of Interest

The authors declare that the research was conducted in the absence of any commercial or financial relationships that could be construed as a potential conflict of interest.

## Publisher's Note

All claims expressed in this article are solely those of the authors and do not necessarily represent those of their affiliated organizations, or those of the publisher, the editors and the reviewers. Any product that may be evaluated in this article, or claim that may be made by its manufacturer, is not guaranteed or endorsed by the publisher.
